# Torsades de Pointe Associated with Trazodone Consumption

**DOI:** 10.1155/2024/5759229

**Published:** 2024-04-20

**Authors:** Hamid Khederlou, Vanoushe Azimi Pirsaraei

**Affiliations:** ^1^Department of Cardiology, Zanjan University of Medical Sciences, Zanjan, Iran; ^2^Student Research Committee, School of Medicine, Zanjan University of Medical Sciences, Zanjan, Iran

## Abstract

**Introduction:**

Trazodone is a serotonin receptor antagonist and reuptake inhibitor commonly used to treat major depression disorder (MDD), anxiety, and sleep disorders. It is considered safe for the heart due to minimal anticholinergic effects. Prolonged QT intervals can cause polymorphic ventricular tachycardia, known as torsades de pointe (TdP). We present a case of a 67-year-old female with a history of MDD who developed trazodone-induced TdP. *Case Presentation*. The patient was referred to a tertiary hospital with a ten-hour history of nausea and vomiting. Trazodone (50 mg daily) was started for her six days ago due to her past medical history of MDD. The initial electrocardiography (ECG) revealed a prolonged corrected QT interval (QTc = 586 ms) due to a long ST segment and generalized T wave inversion. A few moments after admission to the intensive care unit, she suddenly lost consciousness. ECG monitoring showed a TdP, which terminated immediately with the asynchronous defibrillation. A temporary pacemaker was implanted due to repeated arrhythmias and bradycardia. Arrhythmia did not recur for hours and days later. After four days of stopping trazodone, all abnormal ECG findings were resolved, and she was discharged with a normal ECG. She was followed up six months later; the ECG was normal, and she had no complaints.

**Conclusion:**

Trazodone may lead to QTc prolongation and TdP, potentially fatal even without risk factors for QTc prolongation. Close monitoring is essential to prevent adverse complications in trazodone users.

## 1. Introduction

Trazodone is a Food and Drug Administration- (FDA-) approved antidepressant that acts as a serotonin receptor antagonist and reuptake inhibitor commonly used to treat major depression disorder (MDD), anxiety, and sleep disorders. It has a lower anticholinergic effect than other antidepressants, resulting in lower arrhythmogenic effects. Cardiac dysrhythmias associated with trazodone include bradycardia, atrioventricular block, atrial fibrillation, prolonged QT interval, nonsustained ventricular tachycardia (VT), and sustained VT [1, 2].

Prolonged QT is defined as a corrected QT interval (QTc) of over 440 milliseconds (ms) in males and 450 ms in females, categorized as acquired or congenital. Acquired prolonged QT can be caused by drugs such as antiarrhythmic and electrolyte disturbance. Prolonged QT can lead to a lethal polymorphic VT, known as torsades de pointes (TdP) [3, 4]. We present a case with a history of MDD who developed trazodone-induced TdP.

## 2. Case Presentation

The patient, a 67-year-old woman with a history of MDD and anxiety, was referred to a tertiary hospital with a 10-hour history of nausea and vomiting without chest pain, dyspnea, syncope, palpitation, or headache. She had been taking 50 mg of trazodone daily for the past six days.

She denied taking any other medication or alcohol. There was no prior history of cardiac arrhythmias or sudden cardiac death in her or her family, based on the previous medical records.

On presentation to our hospital, vital signs revealed a blood pressure of 108/64 mmHg, a heart rate of 52 beats per minute, and a respiratory rate of 16 breaths per minute. The Glasgow Coma Scale was 15, and the physical examination yielded no significant findings.

**Figure 1:**
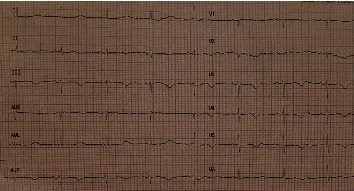
Initial electrocardiography showed sinus bradycardia (rate = 52 beats/minute), normal axis, normal P wave and PR interval, normal QRS voltage and duration, normal R wave progression, and prolonged corrected QT interval (QTc = 586 ms) due to long ST segment and generalized T wave inversion.

The ECG on admission revealed sinus bradycardia (rate = 52 beats per minute) and prolonged QT interval (QTc = 586 ms) due to a long ST segment and generalized T wave inversion. The remaining ECG parameters were within normal limits (Figure 1). She was eventually admitted to the intensive care unit to monitor her vitals and QTc interval continuously.

The initial laboratory workup and basic chemistry panel revealed normal ranges for complete blood count, serum sodium, magnesium, calcium, potassium, chloride, bicarbonate, creatinine, blood urea nitrogen, and fasting blood glucose. Liver and thyroid function tests were normal, and the urinalysis was unremarkable. Abnormal blood levels of acetaminophen, ethanol, and salicylate were not reported, and a comprehensive urine drug screen (done through gas chromatography) was also negative. Unfortunately, we could not obtain a serum trazodone level due to laboratory limitations. Echocardiography confirmed the absence of any cardiac structural, valvular, or functional abnormalities. Echocardiographic findings revealed an ejection fraction of 55%, normal left ventricular size, septal and posterior wall thickness of 9 and 8 mm, no significant valvular heart disease, and normal right ventricular size and function.

**Figure 2:**
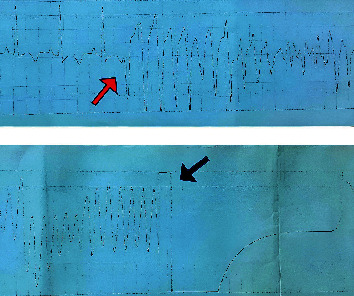
A single long lead strip recording showed torsades de pointes occurred in consent of trazodone overdose (red arrow) that was terminated by asynchronous defibrillation (black arrow).

A few moments after admission, she suddenly lost consciousness. ECG monitoring showed polymorphic wide QRS complex tachycardia, indicating polymorphic ventricular tachycardia, and the patient was pulseless. The arrhythmia terminated immediately with the asynchronous defibrillation (energy shock of 200 joules) (Figure 2). A few minutes later, the arrhythmia recurred, and once again, it was terminated with asynchronous defibrillation (energy shock of 200 joules). A temporary pacemaker was implanted for her due to repeated arrhythmias and bradycardia. A single 2 g dose of intravenous magnesium sulfate was administered over 15 minutes, followed by one gram per hour for 12 hours. Arrhythmia did not recur for hours and days later.

**Figure 3:**
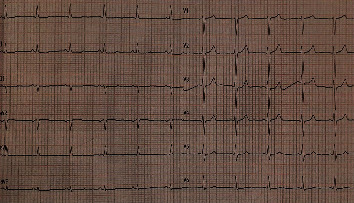
Normal sinus rhythm (rate = 75 beats/minute), normal axis, normal P wave and PR interval, normal QRS voltage and duration, normal R wave progression, and normal QT interval (QTc = 445 ms).

Electrolytes were regularly monitored and remained within normal range throughout her hospitalization. Following consultations with the clinical pharmacist and psychiatrist, trazodone was discontinued. After four days, all abnormal ECG findings were removed, and the patient was transferred from the intensive care unit to a regular medical unit before being discharged home (Figure 3). According to the consultation with the psychiatrist, fluoxetine 20 mg daily was started after stopping trazodone. Subsequent follow-ups at one week, one month, and six months revealed normal ECG results and no complaints.

## 3. Discussion

Trazodone, an antidepressant, was developed in Italy in the 1960s and approved by the FDA in the 1980s. It acts as both a selective serotonin reuptake inhibitor and a 5HT2 receptor antagonist, which is used to treat MDD, anxiety disorders, and difficulties with sleep [5].

Adverse effects of trazodone encompass drowsiness, ataxia, nausea and vomiting, dry mouth, coma, and even death. Cardiac complications manifest as dysrhythmias and hypotension. Cardiac dysrhythmias associated with trazodone include bradycardia, atrioventricular block, atrial fibrillation, prolonged QT interval, nonsustained VT, and sustained VT [5–10]. To date, a few cases of TdP associated with trazodone consumption have been documented [11, 12].

TdP is a potentially lethal polymorphic VT linked to QTc prolongation. Trazodone decreases the maximum upstroke velocity and blocks the delayed rectifier and HERG potassium channels, leading to a prolonged action potential in phase 3. This ultimately results in QT prolongation and polymorphic VT [13, 14]. Similar to the case of the present study, women over 60 years old face an increased risk of drug-induced TdP. The TdP is more likely to occur in cases of electrolyte disturbances, heart failure, and the use of QT-prolonging medications, cytochrome P450 inhibitors, diuretics, digoxin, and methadone. As observed in our case, the QT interval typically returns to normal when the underlying cause is removed [11, 12].

The trazodone level was not measured in this case due to the unavailability of related laboratory measurements in our country. The diagnosis was confirmed based on the absence of a history of arrhythmia, TdP occurrence after taking trazodone, and TdP resolution after stopping the drug. Furthermore, previous studies have shown that trazodone overdose is not correlated with its serum level [13].

## 4. Conclusion

Trazodone may lead to QTc prolongation and TdP, potentially fatal even without risk factors for QTc prolongation. Close monitoring is essential to prevent adverse complications in trazodone users.

## Data Availability

Data is available from the first authors upon request.
